# A combined linkage and regional association mapping validation and fine mapping of two major pleiotropic QTLs for seed weight and silique length in rapeseed (*Brassica napus* L.)

**DOI:** 10.1186/1471-2229-14-114

**Published:** 2014-04-29

**Authors:** Na Li, Jiaqin Shi, Xinfa Wang, Guihua Liu, Hanzhong Wang

**Affiliations:** 1 Oil Crops Research Institute of the Chinese Academy of Agricultural Sciences, Key Laboratory of Biology and Genetic Improvement of Oil Crops, Ministry of Agriculture, Wuhan 430062, China

**Keywords:** Rapeseed (*Brassica napus* L.), Linkage mapping, Regional association mapping, Seed weight/size, Silique length, Fine mapping, Linkage disequilibrium, Pleiotropy

## Abstract

**Background:**

Seed weight (SW) and silique length (SL) are important determinants of the yield potential in rapeseed (*Brassica napus* L.). However, the genetic basis of both traits is poorly understood. The main objectives of this study were to dissect the genetic basis of SW and SL in rapeseed through the preliminary mapping of quantitative trait locus (QTL) by linkage analysis and fine mapping of the target major QTL by regional association analysis.

**Results:**

Preliminary linkage mapping identified thirteen and nine consensus QTLs for SW and SL, respectively. These QTLs explained 0.7-67.1% and 2.1-54.4% of the phenotypic variance for SW and SL, respectively. Of these QTLs, three pairs of SW and SL QTLs were co-localized and integrated into three unique QTLs. In addition, the significance level and genetic effect of the three co-localized QTLs for both SW and SL showed great variation before and after the conditional analysis. Moreover, the allelic effects of the three QTLs for SW were highly consistent with those for SL. Two of the three co-localized QTLs, *uq.A09*-*1* (mean *R*^*2*^ = 20.1% and 19.0% for SW and SL, respectively) and *uq.A09*-*3* (mean *R*^*2*^ = 13.5% and 13.2% for SW and SL, respectively), were detected in all four environments and showed the opposite additive-effect direction. These QTLs were validated and fine mapped (their confidence intervals were narrowed down from 5.3 cM to 1 cM for *uq.A09*-*1* and 13.2 cM to 2.5 cM for *uq.A09*-*3*) by regional association analysis with a panel of 576 inbred lines, which has a relatively rapid linkage disequilibrium decay (0.3 Mb) in the target QTL region.

**Conclusions:**

A few QTLs with major effects and several QTLs with moderate effects might contribute to the natural variation of SW and SL in rapeseed. The meta-, conditional and allelic effect analyses suggested that pleiotropy, rather than tight linkage, was the genetic basis of the three pairs of co-localized of SW and SL QTLs. Regional association analysis was an effective and highly efficient strategy for the direct fine mapping of target major QTL identified by preliminary linkage mapping.

## Background

Linkage and association analyses are two complementary strategies for the genetic dissection of complex quantitative traits. Compared with each other, linkage mapping has relatively high power and a low false positive rate, whereas association mapping has relatively high resolution [1,2]. Linkage mapping is the traditional approach for identifying quantitative trait locus (QTL). Association mapping (including genome-wide, candidate gene and regional association) was originally used in humans [3] and animals [4,5] and has been introduced to plants [6] in recent years. Very recently, joint linkage-association mapping strategies have been proposed to utilize each method [7,8], including parallel mapping (independent linkage and LD analysis) [9-13] and integrated mapping (dataset analysis in combination), such as MAGIC (Multi-parent advanced generation inter-crosses) [14] and NAM (nested association mapping) [15].

Both the seed weight (SW) and silique length (SL) are important determinants of yield potential in rapeseed and are good targets for selection in breeding [16,17] due to their high heritability [18]. The correlation between SW and SL has been investigated by many studies, but the directions of the coefficients were not consistent [19-21]. In general, an increase in silique length may lead to an increase in the source of matter [22], which results in larger seeds. Both SW and SL are quantitatively inherited, which are controlled by multiple QTLs, mainly with additive effects [20,21,23]. Only linkage analysis has been used for mapping QTLs of SW and SL in rapeseed [20,21,24-29], and no association analysis studies have been reported until now.

In particular, neither of the QTLs for SW and SL has been fine mapped. Following preliminary linkage mapping, the classical/traditional fine mapping strategy is based on the recombinant individuals screened from a large-scale NIL (near isogenic lines)-segregating population, which requires several rounds of successive backcrossing and self-crossing (cost of at least two years) and the genotyping of thousands of individuals [30,31]. Thus, the traditional NIL-based fine mapping approach is time-consuming and labor-intensive. As an alternative, because of its relatively high resolution, association mapping can be used for fine mapping. However, high-throughput genome-wide association analysis is unnecessary and wasteful for fine mapping one particular QTL of interest. To overcome these limitations, we proposed a combined linkage and regional association mapping strategy, which conducted association mapping at the specific genomic region of the target QTL that was identified by the preliminary linkage mapping.

In the current study, we used regional association mapping to validate and fine map two major SW and SL QTLs on the A09 linkage group of rapeseed that were identified by the preliminary linkage mapping. In detail, the main objectives of this study were as follows: (1) preliminary mapping of the QTLs for SW and SL using linkage analysis; (2) validation and fine mapping of the target major QTLs using regional association analysis; and (3) determination of the genetic basis of the co-localization of SW and SL QTLs using meta-, conditional and allelic effect analyses.

## Results

### Linkage mapping of the QTLs for SW and SL

#### Phenotypic variation of the parents and segregating populations across environments

**Figure 1:**
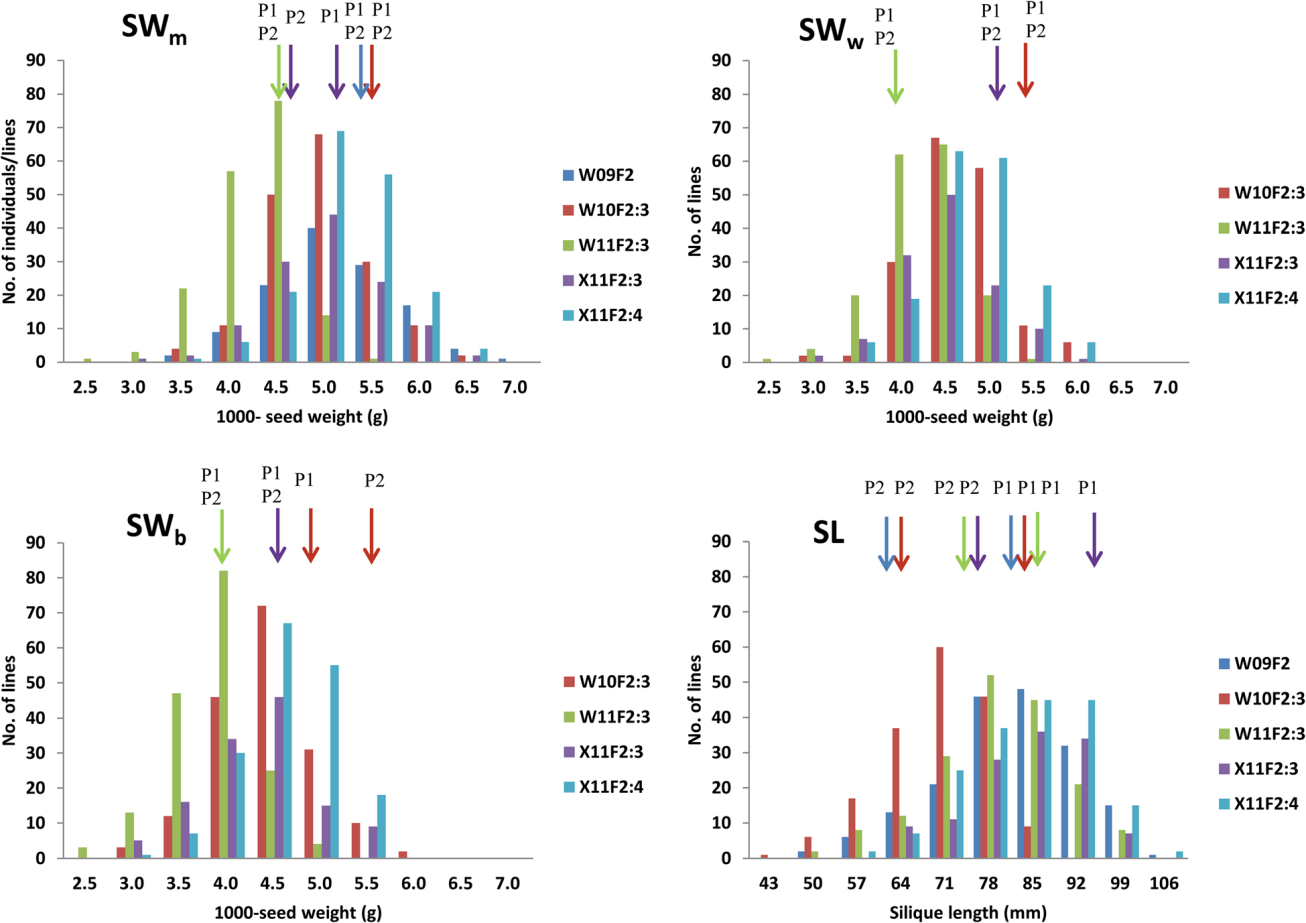
**Distribution of the seed weight and silique length in the F**_**2**_, **F**_**2:3 **_**and F**_**2:4 **_**populations derived from the cross of Zhongshuang11 × No. 73290.** SW_m_, SW_b_ and SW_w_ represent the thousand seed weight of seeds sampled from main raceme, raceme branch, and whole plant, respectively; P1 and P2 indicates Zhongshuang11 and No. 73290, respectively.

The two parents, Zhongshuang11 and No. 73290, differed significantly for SL but not SW in all the investigated environments (Additional file 1: Table S1). Transgressive segregation was observed for all of the populations in all environments, indicating the presence of favorable alleles in both parents. Both the SW and SL of the segregating populations showed normal or near-normal distributions (Figure 1, Additional file 1: Table S1), suggesting a quantitative inheritance pattern suitable for QTL identification. Interestingly, SW_m_ (main raceme thousand seed weight) was higher than SW_b_ (raceme branch thousand seed weight) by approximately 10% for both the parents and all of the populations in all environments, which was in agreement with a previous report [32].

The analysis of variance indicated that the genotypic, environmental and genotype × environment effects were all extremely significant for both SW and SL (Additional file 1: Table S2). Both SW and SL showed very high and similar heritability (*h*^*2*^ = 0.89, 0.88, 0.90 and 0.91 for SW_m_, SW_b_, SW_w_ (whole-plant thousand seed weight) and SL, respectively), which was generally consistent with previous studies [21,27,29].

As expected, highly positive correlations were observed between SW_m_, SW_b_ and SW_w_ in each experiment (Additional file 1: Table S3). A positive correlation between SW and SL was observed with moderate coefficients in almost all of the experiments (Table 1).

**Table 1:** **Pearson**’**s correlation coefficients of seed weight and silique length**

**Experiments code**	**Trait**	**SL**				
		**W09F**_**2**_	**W10F**_**2:3**_	**W11F**_**2:3**_	**X11F**_**2:3**_	**X11F**_**2:4**_
W09F_2_	SW_m_	0.34**	0.26**	0.23*	0.19	0.30**
W10F_2:3_	SW_m_	0.38**	0.46**	0.52**	0.52**	0.46^**^
	SW_b_	0.35**	0.47**	0.49**	0.51**	0.48**
	SW_w_	0.38**	0.48**	0.51**	0.52**	0.48**
W11F_2:3_	SW_m_	0.47**	0.57**	0.62**	0.57**	0.56**
	SW_b_	0.37**	0.47**	0.52**	0.50**	0.53**
	SW_w_	0.43**	0.53**	0.58**	0.56**	0.56**
X11F_2:3_	SW_m_	0.34**	0.39**	0.45**	0.38**	0.30**
	SW_b_	0.34**	0.44**	0.46**	0.44**	0.34**
	SW_w_	0.34**	0.44**	0.48**	0.43**	0.34**
X11F_2:4_	SW_m_	0.36**	0.50**	0.47**	0.45**	0.42**
	SW_b_	0.37**	0.50**	0.47**	0.47**	0.44**
	SW_w_	0.39**	0.52**	0.49**	0.47**	0.46**

“*” and “**” represent the significant level of P = 0.05 and 0.01, respectively.

#### Genome-wide detection and meta-analysis of the QTLs

A framework of the genetic linkage map containing 529 loci (Additional file 1: Table S4) was constructed, which covered a total of 1934 cM of the *B. napus* genome and had an average distance of 3.7 cM between adjacent loci. The segregation distortion of each locus was estimated by the goodness-of-fit test, and 110 loci (20.7%) showed distorted segregation. The biased loci were distributed unevenly: most of them were located on A01, A04, A06, A08, A09, C04 and C08 linkage groups, and the loci biased to the same parent tended to cluster together, which is a common phenomenon in *B. napus*[21,26,33]. Genome-wide QTL analysis was performed for SW and SL separately.

A total of 51 SW identified QTLs (25 significant QTLs and 26 overlapping suggestive QTLs) were detected (Additional file 1: Table S5). Of these, 18, 16 and 17 could be detected for main raceme, raceme branch and whole-plant thousand seed weight, respectively. These identified QTLs explained 0.7 - 67.1% of the phenotypic variance (mean *R*^*2*^ = 12.4%). The meta-analysis integrated 48 overlapping identified QTLs into 10 repeatable consensus QTLs on the A01, A03, A04, A07, A08, A09 and C02 linkage groups (Table 2). Of these, five repeatable consensus QTLs were integrated from different tissues in the same experiment (experiment-specific), and the remaining five were integrated from different experiments (experiment-repeatable). Of the five experiment-repeatable consensus QTLs, *cqSW.A01*-*2* and *cqSW.A07* were detected in two environments (mean *R*^*2*^ = 6.5% and 6.6%, respectively), *cqSW.A08* and *cqSW.A09*-*3* were detected in three environments (mean *R*^*2*^ = 5.3% and 13.5%, respectively), and only one consensus QTL, *cqSW.A09*-*1*, was consistently detected in all four environments (mean *R*^*2*^ = 20.1%).

**Table 2:** **Consensus QTLs for seed weight and silique length obtained by meta**-**analysis**

**Consensus QTL**	**Linkage group**	**LOD**	**R**^**2**^** (%)**	**Peak position**	**Confidence interval (2-LOD)**	**Additive effect**^**a**^	**Experiments code (m, b, w) **^**b**^
*cqSW.A01*-*1*	A01	4.8-5.5	1.7-1.8	32.3	31.2-33.5	-	W11F_2:3_(m,w)
*cqSW.A01*-*2*	A01	2.7-3.5	6.2-6.7	43.9	41.6-46.2	-	W10F_2:3_(b)|W11F_2:3_(b,w)
*cqSW.A01*-*3*	A01	3.7-3.7	0.8-1.7	145.3	144.0-146.6	+	W11F_2:3_(m,b)
*cqSW.A03*-*1*	A03	6.2	15.1	54.2	54.0-55.5	0.42	W09F_2_(m)
*cqSW.A03*-*2*	A03	2.6-3.0	9.1-11.1	79.8	78.7-80.9	+	W10F_2:3_(b,w)
*cqSW.A04*	A04	3.8-4.0	0.7-2.5	76	75.5-76.5	+	W11F_2:3_(m,w)
*cqSW.A07*	A07	2.6-3.6	4.0-11.7	76.2	72.9-79.6	±	W10F_2:3_(b)|W11F_2:3_(m,b,w)
*cqSW.A08*	A08	2.7-5.1	1.1-13.9	22	20.5-23.4	±	W09F_2_(m)|W10F_2:3_(b,w)|W11F_2:3_(b,w)
*cqSW.A09*-*1*	A09	2.7-10.0	9.1-67.1	42	40.9-43.1	-	W09F_2_(m)|W10F_2:3_(m,b,w)|W11F_2:3_(m,b,w)|X11F_2:3_(m)|X11F_2:4_(m,b,w)
*cqSW.A09*-*2*	A09	9.3	13.4	86.3	84.3-88.2	0.34	W10F_2:3_(m)
*cqSW.A09*-*3*	A09	5.8-9.0	7.2-26.9	109.4	106.5-112.3	+	W10F_2:3_(m,b,w)|W11F_2:3_(m,w)|X11F_2:4_(m,b,w)
*cqSW.C02*	C02	3.6-4.4	7.6-1.9	27.2	26.7-27.8	-	W11F_2:3_(m,w)
*cqSW.C06*	C06	4.3	3.4	5	0-10.5	-0.19	X11F_2:4_(m)
*cqSL.A04*-*1*	A04	7.1	5.9	27.8	26.8-28.0	-8.61	X11F_2:3_
*cqSL.A04*-*2*	A04	7.2	20.1	37.5	35.6-39.5	-4.77	X11F_2:3_
*cqSL.A06*	A06	6.2	9	23	17.3-25.6	6.19	X11F_2:4_
*cqSL.A09*-*1*	A09	4.5-7.8	7.8-544	45.1	44.0-46.2	±	W09F_2_|W10F_2:3_|W11F_2:3_|X11F_2:3_|X11F_2:4_
*cqSL.A09*-*2*	A09	10.0-15.4	9.9-16.6	109	102.5-115.6	+	W09F_2_|W10F_2:3_
*cqSL.C01*	C01	2.6-6.6	2.1-9.7	40.2	36.9-43.5	-	W09F_2_|W10F_2:3_|W11F_2:3_
*cqSL.C02*-*1*	C02	4.4	7.7	27.2	25.9-28.7	-9.8	W10F_2:3_
*cqSL.C02*-*2*	C02	4.1	5.1	35.9	31.6-42.2	-3.37	W11F_2:3_
*cqSL.C02*-*3*	C02	5.4	6.3	81.5	80.7-88.0	-4.58	X11F_2:3_

^a^: “+”, “-” and “±” indicate the direction of the additive effect.

^b^: m, b and w represent the main raceme, raceme branch and whole-plant thousand seed weight, respectively.

A total of 18 SL identified QTLs (14 significant QTLs and four overlapping suggestive QTLs) were detected (Additional file 1: Table S5). These identified QTLs explained 2.1 - 54.4% of the phenotypic variance (mean *R*^*2*^ = 12.4%). The meta-analysis integrated 12 overlapping identified QTLs into three repeatable consensus QTLs on the A09 and C02 linkage groups (Table 2). Of the three experiment-repeatable consensus QTLs, *cqSL.A09*-*2* was detected in two environments (mean *R*^*2*^ = 13.2%), *cqSL.C01* was detected in three environments (mean *R*^*2*^ = 4.9%), and only *cqSL.A09*-*1* was detected in all four environments (mean *R*^*2*^ = 19.0%).

The consensus QTLs for SW and SL were subjected to meta-analysis again, which resulted in 19 unique QTLs (Table 3). Of these, three unique QTLs, *uq.A09*-*1*, *uq.A09*-*3* and *uq.C02*-*1* were responsible for both SW and SL. Specially, *uq.A09*-*1* (flanking 5.3 cM region) and *uq.A09*-*3* (flanking 13.2 cM region) were located on the A09 linkage group, with opposite additive-effect directions for both SW and SL.

**Table 3:** **Unique QTLs obtained from the meta**-**analysis of the consensus QTLs for each linkage group**, **separately**

**Unique QTL**	**Linkage group**	**Peak position**	**Additive effect**	**Type**
*uq.A01*-*1*	A01	32.3	-	SW-specific
*uq.A01*-*2*	A01	43.9	-	SW-specific
*uq.A01*-*3*	A01	145.3	+	SW-specific
*uq.A03*-*1*	A03	54.2	0.42	SW-specific
*uq.A03*-*2*	A03	79.8	+	SW-specific
*uq.A04*-*1*	A04	27.8	-8.61	SL-specific
*uq.A04*-*2*	A04	37.5	-4.77	SL-specific
*uq.A04*-*3*	A04	76.0	+	SW-specific
*uq.A06*	A06	23.0	6.19	SL-specific
*uq.A07*	A07	76.2	±	SW-specific
*uq.A08*	A08	22.0	±	SW-specific
*uq.A09*-*1*	A09	41.8	-	Pleiotropic
*uq.A09*-*2*	A09	86.3	0.34	SW-specific
*uq.A09*-*3*	A09	109.3	+	Pleiotropic
*uq.C01*	C01	40.2	-	SL-specific
*uq.C02*-*1*	C02	27.2	-	Pleiotropic
*uq.C02*-*2*	C02	35.9	-3.37	SL-specific
*uq.C02*-*3*	C02	81.5	-4.58	SL-specific
*uq.C06*	C06	5.0	-0.19	SW-specific

“+”, “-” and “±” indicate the direction of the additive effect.

To determine the genetic basis of three unique QTLs for both SW and SL (pleiotropy or tight linkage), conditional QTL analysis was performed (Table 4). When SW (represented by SW_m_) was conditioned by SL (SW_m_|SL), none of the three loci (*uq.A09*-*1*, *uq.A09*-*3*, and *uq.C02*-*1*) remained significant for SW in all experiments; when SL was conditioned by SW (SL|SW_m_), these loci were not significant for SL in half of the experiments. These results strongly suggested that pleiotropy, rather than tight linkage was likely to be the genetic cause of the three unique QTLs for both SW and SL, and that SW was possibly contributed by SL for these loci.

**Table 4:** Conditional analysis for the unique QTLs identified by linkage mapping

**Unique QTL**	**Experiments code**	**Additive effect/R**^**2 **^**(%)**			
		**SW**_**m**_^**a**^	**SW**_**m**_**|SL**^**b**^	**SL**	**SL|SW**_**m**_
	W09F_2_	-0.22/11.7		-6.13/54.4	-5.58/30.2
	W10F_2:3_	-0.31/29.5		-3.64/15.2	
*uq.A09*-*1*	W11F_2:3_	-0.18/16.9		-3.42/12.3	-6.89/2.3
	X11F_2:3_	-0.17/15.1		-4.23/19.6	
	X11F_2:4_	-0.20/18.9		-3.18/11.7	
*uq.A09*-*3*	W09F_2_			9.87/16.6	7.71/15.1
	W10F_2:3_			3.52/4.8	2.40/3.2
	W11F_2:3_	0.33/11.0			
	X11F_2:4_	0.30/7.9			
*uq.C02*-*1*	W10F_2:3_			-9.80/7.7	
	W11F_2:3_	-0.92/1.9			

^a^: Only the main raceme 1000 seed weight dataset is used in each experiment for the conditional analysis.

x|y^b^: Indicates trait x is conditioned by trait y.

### Regional association mapping

#### SSR (Single Sequence Repeat) markers used for association mapping

**Figure 2:**
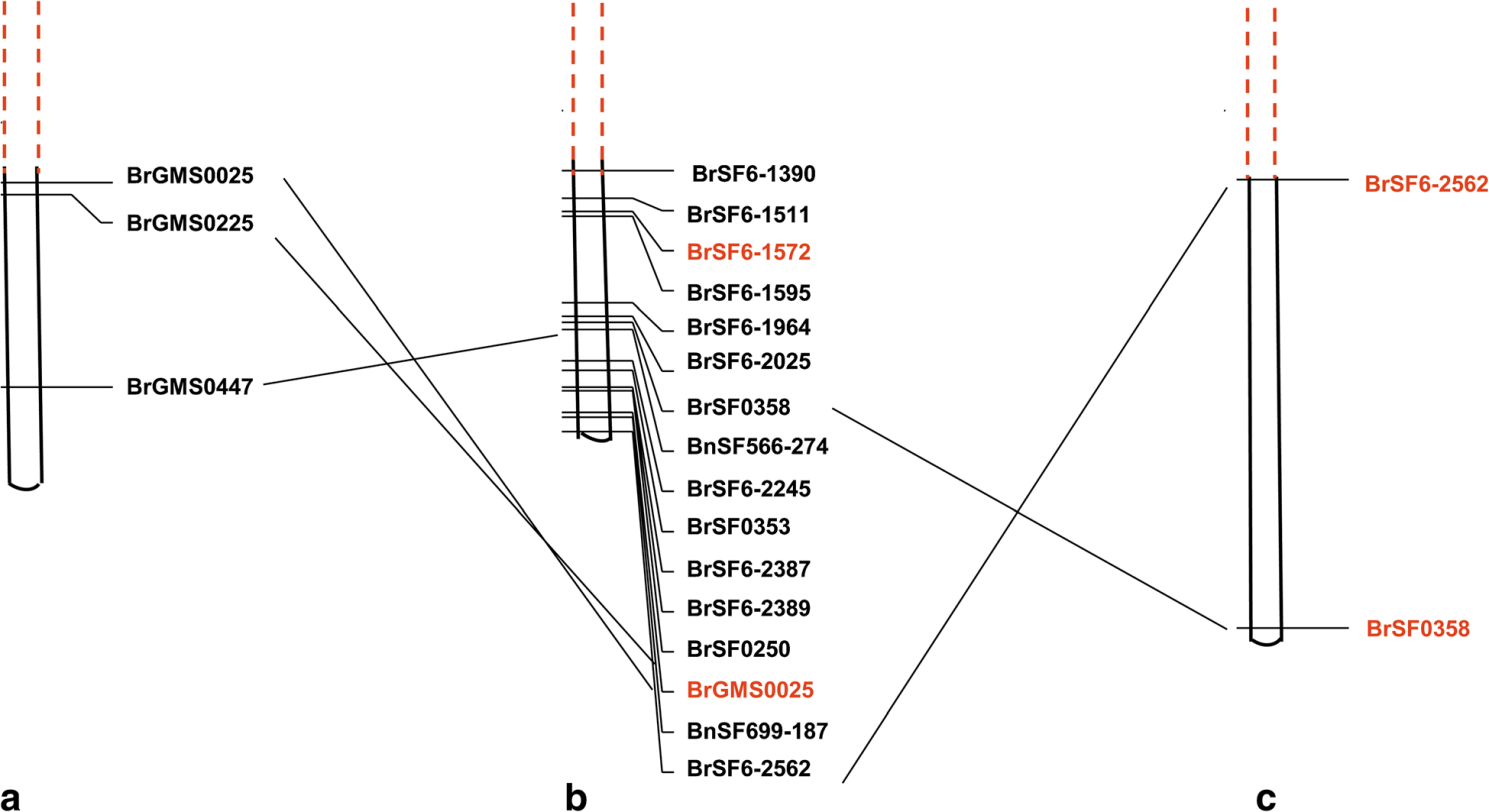
**Integration of the physical and genetic maps in the target QTL region. a**: the markers in the order of the genetic map (cM) for *B. napus* based on a previous study (Xu et al. 2010); **b**: the markers in the order of the physical map (Kb) for *B. rapa*. The markers in red are the most associated markers for SW and SL; **c**: the markers in the order of the genetic map (cM) for *B. napus* in the current study. The markers in red are the nearest markers to the two unique QTLs for SW and SL. The dashed red line represents the other region of the map.

The corresponding genomic regions of two major unique QTLs (*uq.A09*-*1* and *uq.A09*-*3*) were identified by the alignment between the primer sequences of tightly linked SSR markers (BrSF6-2562 and BrSF0358) and the genomic sequences of *B. napus* (unpublished data) and *B. rapa*[34] due to the macro-colinearity between the A genomes of *B. rapa* and *B. napus*[35]. In total, 108 and 106 SSR markers (Additional file 1: Table S6) within the corresponding genomic regions of the two QTLs were newly synthesized. Of these, both six primer pairs were polymorphic between the two parents in the linkage mapping, and five and three SSR markers were selected for each locus for the association mapping. To screen more SSR markers for association mapping, the mini-core germplasms (Zhongshaung11, No. 73290, Tapitor and No. 91550) were used to screen the polymorphisms for the other SSR markers (including newly synthesized SSR markers and published SSR markers), and we obtained three and six polymorphic primer pairs for the two unique QTLs (Figure 2).

#### Regional association mapping

A large range of phenotypic variations was observed (Additional file 1: Table S1) for both SW (~4-fold) and SL (~3-fold) in the association population. A significant weak correlation (0.47) was observed between SW and SL.

**Figure 3:**
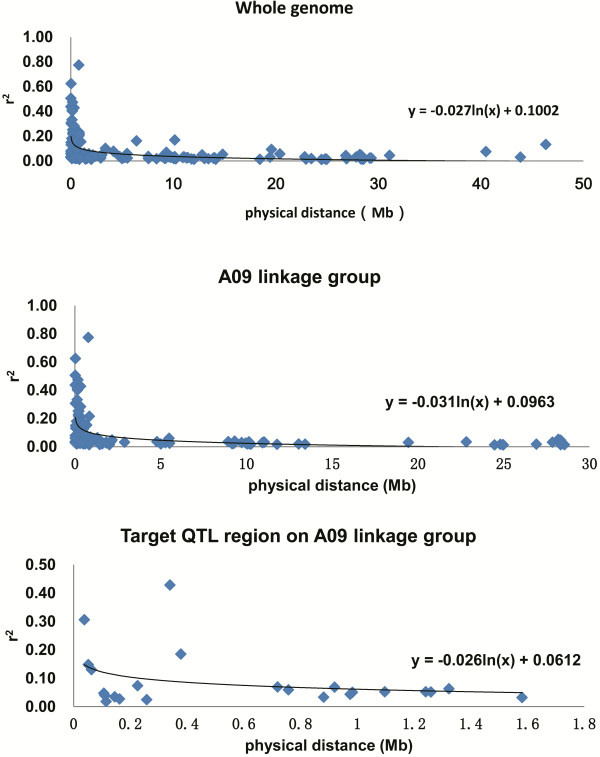
**Scatterplot of the significant LD (r**^**2**^**) against physical distance (Mb) for the whole genome, A09 linkage group and target QTL region.**

In this study, the 95^th^ percentile of the *R*^*2*^ distribution for unlinked markers (markers from different chromosomes, Additional file 1: Table S7) determined the background level of LD (*R*^2^ < 0.091). The extent of the LD decay was evaluated using linked markers (markers from the same chromosomes). The LD decay decreased within 1.40 Mb over the whole genome and within 1.19 Mb on the A09 linkage group. In particular, the extent of the LD decay for the target QTL region (major QTLs, discarding the markers involved in inversion, Figure 2) was 0.33 Mb (Figure 3).

**Figure 4:**
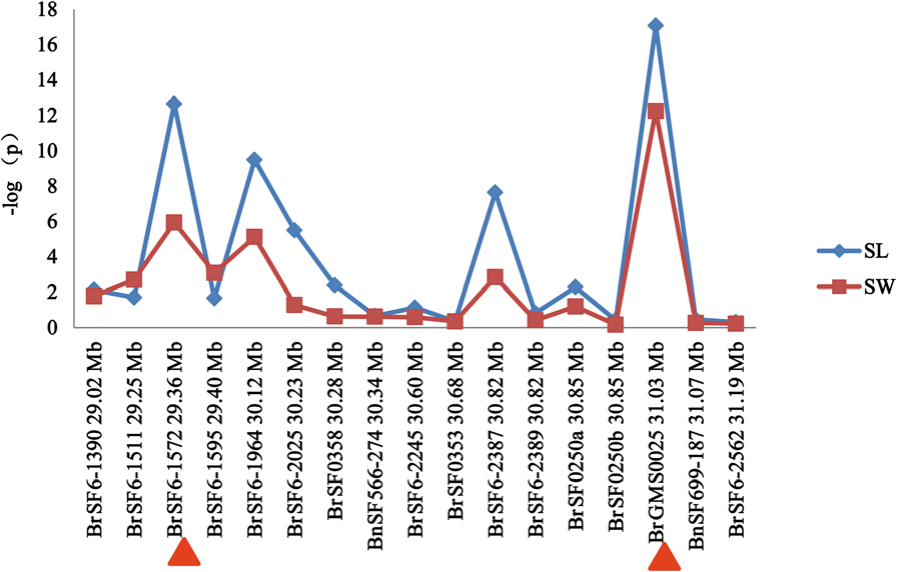
**Scanning of the association (in -log10[p]) of seed weight and silique length with 17 marker loci on the A09 linkage group in rapeseed.** The 17 marker loci are ordered on the horizontal axis according to their physical positions on the A09 linkage group of *B. rapa*. The red arrow points to peak signals.

Considering the population structure (Additional file 1: Tables S7 and S8) and family relatedness (Additional file 1: Table S9) within the population, the association analysis was conducted with a mixed linear model (MLM) by TASSEL 3.0 using the 576-line sets and 17 QTL-linked SSR loci in the target region (Additional file 1: Table S6). Notably, six and eight of the 17 loci on the A09 linkage group (Table 5) with lower p-values (significant) were identified for SW and SL, respectively. Scanning of the association of SW and SL with the 17 loci on the A09 linkage group generally displayed two obvious peaks (Figure 4), which corresponded to the abovementioned two unique QTLs, *uq.A09*-*1* and *uq.A09*-*3*. Within the first peak, the marker BrGMS0025 showed the strongest association for both SW (p = 5.7E-13; *R*^*2*^ = 14.6%) and SL (p = 8.4E-18; *R*^*2*^ = 18.8%) and was very near to BrSF6-2562, the nearest marker for *uq.A09*-*1*. Within the second peak, the marker BrSF6-1572 showed the strongest association signal for both SW (p = 1.2E-6; *R*^*2*^ = 7.2%) and SL (p = 2.2E-13; *R*^*2*^ = 13.8%) and was near to BrSF0358, the nearest marker for *uq.A09*-*3*.

**Table 5:** Association and conditional analysis for seed weight and silique length

**Marker**	**Linkage group**	**Positions (Mb)**	**p value/R**^**2 **^**(%)**					
			**SW**	**SWSL1**^**a**^	**SWSL2**^**b**^	**SL**	**SLSW1**	**SLSW2**
BrSF6-1390	A09	29.02				7.7E-03/5.5		
BrSF6-1511	A09	29.25	2.0E-03/4.3					
BrSF6-1572	A09	29.36	1.2E-06/7.2			2.2E-13/13.8	2.5E-06/7.0	
BrSF6-1595	A09	29.4	8.2E-04/4.2					2.2E-07/6.8
BrSF6-2245	A09	30.6						1.6E-04/6.1
BrSF6-1964	A09	30.12	7.7E-06/8.6			3.3E-10/14.9	1.0E-03/6.2	
BrSF6-2025	A09	30.23				3.3E-06/8.8	2.9E-04/7	1.6E-04/6.1
BrSF0358	A09	30.28				4.2E-03/3.9		
BrSF6-2387	A09	30.82	1.4E-03/7.2			2.3E-08/12.8	5.0E-05/9.4	
BrSF6-2389	A09	30.82						1.2E-05/8.6
BrSF0250a	A09	30.85				5.3E-03/2.7		
BrGMS0025	A09	31.03	5.7E-13/14.6	5.5E-03/3.2	1.8E-06/6.3	8.4E-18/18.8	1.1E-07/8.5	4.6E-10/9.5

x|y1^a^: Indicates that trait x is conditioned by trait y using the first conditional analysis method.

x|y2^b^: Indicates that trait x is conditioned by trait y using the second conditional analysis method.

**Figure 5:**
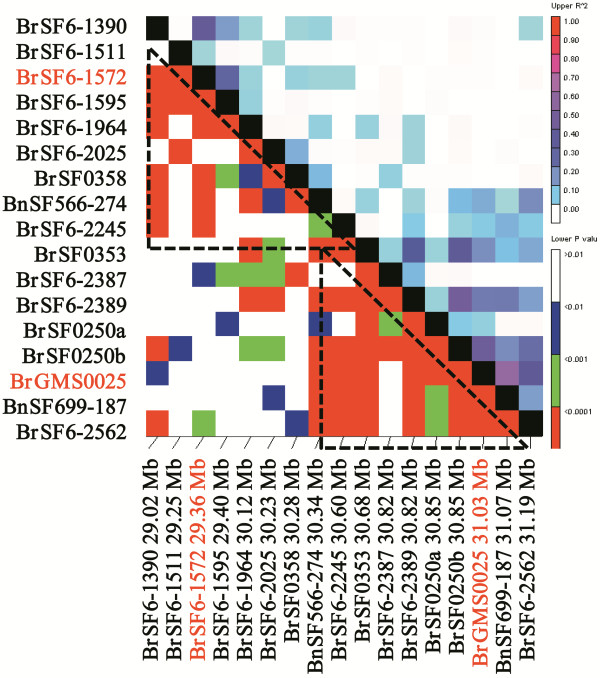
**Local LD map for target QTL region on the A09 linkage group.** The significant level of linkage disequilibrium between each marker pair is indicated below the diagonal; above the diagonal, the level of linkage disequilibrium is indicated. The markers in red are the peak signals.

To determine the resolution of this association study, the extent of the LD around the best associated SSR markers (BrGMS0025 and BrSF6-1572) was investigated. As expected, this region was divided into two LD blocks [36]. Eight and seven markers showed significant LD with BrGMS0025 and BrSF6-1572, respectively (Table 6, Figure 5). Of these, BnSF566-274 and BrSF6-2245 displayed significant LD with both BrGMS0025 and BrSF6-1572, but their *R*^2^ values were relatively lower than those of the other markers, which likely represented the overlapping region of the two LD blocks. The first LD block around the marker BrGMS0025 extended roughly from BrSF0353 (at 30.68 Mb) to BrSF6-2562 (at 31.19 Mb), indicating a resolution of approximately 1 cM (0.51 Mb). Another LD block around the marker BrSF6-1572 extended roughly from BrSF6-1390 (at 29.02 Mb) to BrSF0358 (at 30.28 Mb), indicating a resolution of approximately 2.5 cM (1.26 Mb).

**Table 6:** **Pairwise LD estimates between the peak signals**, **BrSF6**-**1572 and BrGMS0025**, **with the other markers at the level of p** ≤ **0.001**

**Peak signals**	**Position (Mb)**	**Other markers**	**Position (Mb)**	**Distance (Mb)**	**r**^**2**^	**p value**
BrSF6-1572	29.36	BrSF6-1390	29.02	0.34	0.43	0
BrSF6-1572	29.36	BrSF6-1511	29.25	0.11	0.05	7.8E-07
BrSF6-1572	29.36	BrSF6-1595	29.40	0.04	0.31	0
BrSF6-1572	29.36	BrSF6-1964	30.12	0.76	0.06	2.1E-06
BrSF6-1572	29.36	BrSF0358	30.28	0.92	0.07	2.2E-07
BrSF6-1572	29.36	BnSF566-274	30.34	0.98	0.05	3.1E-07
BrSF6-1572	29.36	BrSF6-2245	30.60	1.24	0.05	7.1E-07
BrGMS0025	31.03	BnSF566-274	30.34	0.69	0.15	0
BrGMS0025	31.03	BrSF6-2245	30.60	0.43	0.09	0
BrGMS0025	31.03	BrSF0353	30.68	0.35	0.21	0
BrGMS0025	31.03	BrSF6-2389	30.82	0.21	0.25	0
BrGMS0025	31.03	BrSF0250a	30.85	0.18	0.07	0
BrGMS0025	31.03	BrSF0250b	30.85	0.18	0.47	0
BrGMS0025	31.03	BnSF699-187	31.07	0.04	0.62	0
BrGMS0025	31.03	BrSF6-2562	31.19	0.16	0.41	0

#### Conditional analysis

To determine the genetic basis (pleiotropy or tight linkage) of the common association markers for SW and SL, conditional analysis was performed using two methods. The first method used the conditional phenotypic values, while the second method used one trait as a covariate for the other, to perform the association analysis. The results showed that the p value and *R*^2^ of the association markers showed great variation before and after the conditional analysis using both methods (Table 5). Taking one of the peak signal markers, BrGMS0025, as an example, regardless of whether SW was conditioned by SL (SW|SL) or SL was conditioned by SW (SL|SW), both showed a strongly reduced effect (at least seven and eight orders of magnitude, respectively). This result indicated that the genetic basis of common association markers for SW and SL was likely to be pleiotropy rather than tight linkage.

### Allelic effects of the three pairs of co-localized SW and SL QTLs in the linkage and association populations

The allelic effects of the co-localized SW and SL QTLs in the linkage and association populations were estimated using the phenotypic values of the different genotypes for the nearest marker (Table 7, Additional file 1: Table S10). The results showed that for all of the haplotypes of the three co-localized SW and SL QTLs (*uq.A09*-*1*, *uq.A09*-*3* and *uq.C02*-*1*), their allelic effects for SW were highly accordant with those for SL in both the linkage and association populations. For example, the corresponding phenotypic values of the three major haplotypes (A, E and C) of the marker BrSF6-1572 (nearest to *uq.A09*-*3*) for SW and SL were 4.42 g and 65.39 mm, 4.11 g and 62.51 mm, and 3.97 g and 60.41 mm, respectively. This finding increased the likelihood that pleiotropy rather than tight linkage was the underlying genetic basis for the three pairs of co-localized SW and SL QTLs.

**Table 7:** **Effect estimates of the three co**-**localized seed weight and silique length QTL in the linkage and association population**

**Unique QTL**	**Population**	**Marker**	**Genotype**^**a**^	**Sample number**	**SW**_**m**_^**d**^	**SW**_**b**_	**SW**_**w**_	**SL**
*uq.A09*-*1*	linkage mapping	BrSF6-2562	P1 type	58	4.63 ± 0.56a^e^	4.14 ± 0.49a	4.35 ± 0.52a	77.3 ± 10.5a
			P2 type	40	4.88 ± 0.45b	4.33 ± 0.43b	4.56 ± 0.44b	79.8 ± 7.1a
	association mapping	BrGMS0025	D (P1/P2) ^b^	204	4.37 ± 0.95a			66.3 ± 13.5a
			C	275	4.02 ± 0.81b			60.9 ± 10.9b
			A^c^	6				
			B	17				
*uq.A09*-*3*	linkage mapping	BrSF0358	not P2 type	121	4.94 ± 0.39a	4.40 ± 0.37a	4.64 ± 0.37a	82.6 ± 6.4a
			P2 type	63	4.52 ± 0.50b	44.03 ± 0.47b	4.23 ± 0.48b	71.8 ± 7.5b
	association mapping	BrSF6-1572	A	65	4.42 ± 0.93a			65.4 ± 13.0a
			E (P1/P2)	248	4.11 ± 0.85b			62.5 ± 12.6b
			C	171	3.97 ± 0.80bc			60.4 ± 9.2ab
			D	4				
			B	5				
*uq.C02*-*1*	linkage mapping	BoSF1827	not P2 type	114	4.80 ± 0.45a	4.27 ± 0.39a	4.49 ± 0.41a	78.9 ± 7.8a
			P2 type	45	4.85 ± 0.43a	4.32 ± 0.44a	4.57 ± 0.45a	80.1 ± 9.1a

^a^: In linkage mapping, “P1 type” indicates marker phenotype that is the same as that of Zhongshuang11, “P2 type” indicates marker phenotype that is the same as that of No. 73290, “not P2 type ” indicates marker phenotype that is not No. 73290 type; in association mapping, alleles are arranged in alphabetical order according to amplified fragment size. ^b^: “P1/P2” indicates that Zhongshuang11 and No. 73290 have the same genotype in association population.

^c^: Rare alleles with an allele frequency of < 0.05 are treated as missing data in the association population.

^d^: SW_m_, SW_b_ and SW_w_ are the mean values from all the experiments, and the details of each experimental analysis are shown in Additional file 1: Table S10.

^e^: Being followed by the same letter indicates no significant difference at the 0.05 probability level based on a Duncan-test.

## Discussion

In the present study, we proposed a combined linkage and regional association mapping strategy to directly fine map target major QTLs. Using this strategy, the confidence intervals of the two major QTLs on the A09 linkage group were narrowed to approximately 1/5 of those in the preliminary linkage mapping (basically, this strategy was used to achieve fine mapping). Our results suggested that this strategy is effective for direct fine mapping after preliminary linkage analysis. Compared with the traditional/classical NIL-based fine mapping approach [31], this strategy does not require the development and genotyping of a large-scale NIL segregating populations and is time- and labor-saving. In addition, our strategy can be applied to all plant species, especially those lacking high-density genome-wide genetic markers.

In previous genetic and QTL mapping studies, seed weight was usually measured separately from the main raceme (SW_m_), branch raceme (SW_b_) [32] and whole plant (SW_w_) [21,27,29]. In the present work, SW_m_, SW_b_ and SW_w_ were all measured for both the genetic and QTL analyses. Strikingly, SW_m_ showed an extremely high correlation with both SW_b_ (mean r = 0.93) and SW_w_ (mean r = 0.96), and most of the QTLs identified for SW_m_, SW_b_ and SW_w_ were consistent. However, SW_m_ is more easily measured than SW_b_ and SW_w_. We therefore suggested the measurement of SW_m_ rather than SW_b_ and SW_w_ in futher studies.

In the previous linkage QTL mapping studies, approximately 120 and 30 QTLs have been identified for SW [20,21,24,25,27-29,37] and SL [20,21,25,26], respectively, which were distributed on all and 16 of the 19 total linkage groups. Most of these QTLs showed relatively small effects, with only three major QTLs [27]: two on the A07 linkage group for SW and one on the A09 linkage group for both SW and SL. Of the 13 SW and 9 SL consensus QTLs identified in the current linkage and association mapping studies, two (*cqSW.A07* and *cqSW.A09*-*3*) and three (*cqSL.A09*-*2*, *cqSL.C01* and *cqSL.C02*-*3*), respectively, have also been confirmed by the previous studies. The currently identified consensus QTLs, *cqSW.A07*, *cqSL.C01* and *cqSL.C02*-*3*, likely corresponded with *TSWA7a*, *sl11* and *qSL.N12*, respectively, which were detected in one of the previous studies and are located around the common markers BRMS036 [29], CB10369 [26] and CB10026 [20], respectively. The consensus QTLs, *cqSW.A09*-*3* and *cqSL.A09*-*2*, were very close (<1 Mb) to the *cqSWA9* and *cqSLA9* QTLs, respectively, which were identified in a previous study [21]. In addition, six SW QTLs have also been identified repeatedly around the markers CB10597 [21,28,37], MR119 [27,28,37], sR0282R [21,27,29], CB10536 [28,37], Na12E04 [28,37] and Ni4A07 [28,37] on A01, A05, A07, C01, C02 and C09 linkage groups, respectively, according to various previous studies. These “repeatable” QTLs found across the current and previous studies should be potential targets for marker-assisted selection. The four SW and two SL major QTLs found across these studies would be the important targets for map-based cloning. These results showed that both SW and SL were controlled by a large number of loci, mostly with small effects, which strongly suggested the complexity of the genetic basis of both traits.

The allotetraploid *B. napus* (AACC) was derived from chromosome doubling after the recent (~0.01 million years ago) natural hybridization between its two diploid ancestors, *B. rapa* (AA) and *Brassica oleracea* (CC) [38]. The previous comparative genomics studies showed that although most of the homoeologous A genome linkage groups/chromosomes of *B. rapa* and *B. napus* showed co-linearity [35,39], some small-scale genomic changes also existed, including translocations [40], insertion/deletions, inversions and rearrangements [35,41]. In the current study, a large fragment inversion was also revealed by the comparison between the *B. napus* linkage map and the *B. rapa* physical map of the QTL interval of *uq.A09*-*1* and *uq.A09*-*3* (Figure 2), which was also consistent with the previous comparative genomics studies [35]. These results explained the inconsistency between the large genetic distance (30 - 50 cM) of *uq.A09*-*1* and *uq.A09*-*3* in *B. napus* and the close physical distance (<1 Mb) in *B. rapa*.

The estimated genome-wide LD decay of the current *B. napus* association population was 1.4 Mb, which corresponds with approximately 2.8 cM [42,43] and was slightly higher than those estimated (0.5 - 2.0 cM) in previous studies on rapeseed [43-46]. The estimated LD decay on the A09 linkage group was 1.2 Mb, corresponding with 2.4 cM, which was very near that on the whole genome in our study and was slower than that (1 cM) estimated for the same linkage group in a previous study [44]. However, the LD decay of the target QTL interval (0.3 Mb) was faster than those of the A09 linkage group and the whole genome. This observation suggested that the QTL region should be within a recombination hotspot, which was consistent with its location on the end of the A09 pseudo-chromosome and linkage group, most likely corresponding with the peri-telomere. This result also indicated that the target QTLs could be fine mapped through LD mapping with the current association population.

From the linkage and association analyses, a total of three co-localized SW and SL QTLs were identified, with the same additive-effect direction, which agreed with the significantly and moderately positive correlations in both populations. In fact, the co-localization of the SW and SL QTLs was also commonly observed in other previous studies [20,21,27,29]. However, the underlying genetic basis (pleiotropy or tight linkage) has not yet been studied intensively. Interestingly, the allelic effect, conditional and meta-analyses of the three co-localized QTLs all supported that pleiotropy rather than tight linkage was likely to be the underlying genetic basis in the current study. In addition, thousand seed weight of the F_6_ lines with extremely large (SW > 6.0 g) and small (SW < 3.0 g) seeds were in high accordance with the silique length of the corresponding lines (r = 0.87, p < 0.001). Thus, the variations in SW might be primarily affected by those in the SL in the current linkage population, which is in accordance to the abovementioned conditional analysis for the three co-localized QTLs for both SW and SL. This finding is understandable because long siliques enable an increased photosynthesis area and assimilation, thereby providing the basis for the increase in the SW, and implicating maternal control of the underlying gene (s) [47-49]. Therefore, relevant genes within the genomic regions of the two major pleiotropic QTLs for SL rather than SW should be chosen as the preferential candidates. These results shed new light on the screening of candidate genes underlying complex quantitative traits: which one is the causal or the intermediate trait for complex trait?

## Conclusions

In the present study, we proposed a regional association mapping strategy to directly fine map the target QTLs identified in preliminary linkage mapping. Compared with the traditional/classical NIL-based fine mapping strategies, our approach has many advantages, for example, it is time-saving, labor-saving and cost-effective. Using this strategy, the confidence intervals of the two major QTLs for both SW and SL on the A09 linkage group were successfully narrowed to a large extent, demonstrating the effectiveness of our strategy. Interestingly, the meta-, conditional and allelic effect analyses all suggest that pleiotropy, rather than tight linkage, was the genetic basis of the three unique QTLs for both SW and SL. Furthermore, the variations in SL are more likely to be the cause of the variation in SW, not vice versa. These results provide a solid basis for candidate gene screening and further gene cloning. In addition, several SW and/or SL QTLs identified by the current linkage mapping appeared to be “repeatable” in previous studies and could be the potential targets for marker-assisted selection in rapeseed breeding.

## Methods

### Plant materials, field experiments and trait evaluation

The linkage population included 184 F_2_, F_2:3_ and F_2:4_ individuals/lines that were derived from two sequenced rapeseed cultivars, Zhongshuang11 (de novo sequencing, unpublished) and No. 73290 (re-sequencing, unpublished). The association population consisted of a panel of 576 rapeseed inbred lines (Additional file 1: Table S8), including both parental lines in linkage analysis.

Location-year combinations were treated as environments, and environment-population combinations were treated as experiments. The experiments were performed in two contrasting environments (semi-winter and spring rapeseed area). Details of the climate conditions during the growing season are described in Additional file 2: Figure S1. The F_2_ individuals were planted in Wuhan (Hubei province, semi-winter rapeseed area) from Oct. 2008 to May 2009 (code W09F_2_). The F_2:3_ lines were planted in Wuhan from Oct. 2009 to May 2010 (code W10F_2:3_) and Oct. 2010 to May 2011 (code W11F_2:3_) and in Xining (Qinghai province, spring rapeseed area) from April to Aug. 2011 (code X11F_2:3_). The F_2:4_ lines were planted in Xining from April to Aug. 2011 (code X11F_2:4_). The association population was planted in Wuhan from Oct. 2011 to May 2012 (code W12AP).

Both the linkage (including both parents) and association populations were arranged in a randomized complete block design with three replications (except F_2_ individuals). Each block contained two rows with 15 plants per row with spacing of 33.3 × 16.7 cm. The seeds were sown by hand, and the field management followed standard agriculture practice. In each block, 10 representative individuals from the middle of each row were harvested by hand at maturity.

For the linkage populations, the seeds from the main raceme and branch raceme were threshed separately. The SW was measured based on 1000 fully developed seeds. The main raceme thousand seed weight (SW_m_), raceme branch thousand seed weight (SW_b_) and whole-plant thousand seed weight (SW_w_) were each evaluated. For the F_2_ individuals, only SW_m_ was measured. For the F_2:3_ and F_2:4_ lines, SW_m_, SW_b_ and SW_w_ were measured. The SL was measured based on 10 well-developed siliques (not including the beak) from the main raceme. For the association population, SW_m_ and SL were measured using the same method described above.

### Statistical analysis

The broad-sense heritability was calculated as *h*^*2*^ = σ^2^_g_ / (σ^2^_g_ + σ^2^_ge_ / n + σ^2^_e_ / nr), where σ^2^_g_ was the genetic variance, σ^2^_ge_ was the interaction variance of the genotype with environment, σ^2^_e_ was the error variance, n was the number of environments and r was the number of replications. The estimation of σ^2^_g_, σ^2^_ge_ and σ^2^_e_ were obtained from the SAS ANOVA procedure. Pearson’s correlation coefficients were calculated using the SAS CORR procedure based on environment means.

### Polymorphism screening and map construction

Genomic DNA was extracted from leaf tissues of the two parents (Zhongshuang11 and No. 73290) and its derived 184 F_2_ individuals. Three groups of markers from different sources were used for polymorphism screening between the two parents. The first group, mainly consisted of SSR and STS (sequence tagged site) markers, were selected from database of publish molecular markers in *Brassica* (http://www.brassica.info/resource/markers/ssr-exchange.php) and published papers [33,35,50-60]. The second group, all consisted of SSR markers (prefixed “BoSF” and “BrSF”), were developed from three recently sequenced *Brassica* crops (*B. rapa*, *B. oleracea* and *B. napus*) by our lab [61]. The third group consisted of SNP (Single nucleotide polymorphism, prefixed “snap” and “ns”) and InDel (Insertion/Deletion, prefixed “ni”) markers, were also developed from the re-sequencing of No. 73290 by our lab (the primer sequences were provided in Additional file 1: Table S7). For markers detected at more than one polymorphism locus, a lowercase alphabetic letter was added to distinguish the loci. The PCR procedure, electrophoresis and silver staining were performed as described by Shi et al. [61].

The genetic linkage map was constructed using the JoinMap 4.0 software (http://www.kyazma.nl/index.php/mc.JoinMap) with the threshold for goodness-of-fit of ≦ 5, a recombination frequency of < 0.4 and minimum logarithm of odds (LOD) score of 2.0. All genetic distances were expressed in centimorgans (cM) as derived by the Kosambi function [62]. The segregation of each marker in the F2 population was analyzed by a chi-square test for “goodness-of-fit” to an expected segregation ratio (P < 0.01).

### Linkage QTL mapping and meta-analysis

The linkage mapping of QTLs was performed separately for SW and SL using the composite interval mapping program [63] of the WinQTL cartographer 2.5 software (http://statgen.ncsu.edu/qtlcart/WQTLCart.htm). A forward-backward stepwise regression following model 6 was performed to choose the co-factors (chosen with P_in_ = 0.05 and P_out_ = 0.05) before the QTL detection. The control marker numbers, window size and walking speed were set to 5, 10 cM and 1 cM, respectively. A default genetic distance of 5 cM was used to define a QTL in a specific experiment. The experience-wise LOD threshold was determined by a permutation test of 1000 repetitions [64]. LOD scores corresponding to P = 0.05 were used to identify significant QTLs (3.62 - 4.94 for SW and 4.03 - 4.44 for SL). To avoid missing QTLs with very small effects, a lower LOD value corresponding to P ≤ 0.50 was adopted in the presence of suggestive QTLs (2.43 - 3.70 for SW and 2.56 - 3.02 for SL). The overlapping suggestive QTLs and all significant QTLs were admitted [27,65] and named as identified QTLs.

Meta-analysis was used to estimate the number and positions of the meta-QTLs underlying the QTLs of the same or related traits, which were repeatedly detected in different environments and located on the same chromosomal region [66]. The computation was conducted using BioMercator 2.1 software [67]. QTLs repeatedly identified for the same trait in different environments were first integrated into the consensus QTL, and then, the QTLs for different traits were further integrated into the unique QTL.

Each identified QTL was designated with the initial letter “q”, followed by the name of the trait abbreviation (SW/SL) and linkage group. Each consensus QTL was designated with the initial letters “cq”, followed by the name of the trait abbreviation and linkage group. Each unique QTL was designated with the initial letters “uq”, followed by the name of the linkage group. Arabic numerals were added if more than one QTL was located on the same linkage group.

### Conditional QTL analysis

To dissect the genetic basis (pleiotropy or tight linkage) of the co-localization of the SW and SL QTLs, conditional analysis was performed. The conditional phenotypic values y _(T1/T2)_ were obtained by the mixed model approach for the conditional analysis of quantitative traits [68] using QGAStation 1.0 (http://ibi.zju.edu.cn/software/qga/index.htm), where T1|T2 indicates that trait 1 is conditioned by trait 2. Then, the conditional mapping of the QTLs was conducted according to the conditional phenotypic values using the same method as the unconditional QTLs mentioned above.

### Linkage disequilibrium (LD) evaluation

The *R*^2^ value of LD and the corresponding significance level (p value) were calculated using the TASSEL 3.0 standalone software (http://www.maizegenetics.net/index.php?option=com_content&task=view&id=89&Itemid=119) with a permutation test of 1000 repetitions. Rare alleles with an allele frequency of < 0.05 were treated as missing data [69]. The loci were considered to be in significant LD if P ≤ 0.01. The threshold of significant LD for linked loci was defined as the 95% quantile of the *R*^2^ value among unlinked loci pairs [44,70]. Loci on the same linkage group were used to evaluate LD decay. To obtain a better visual description of the LD decay with distance, LD decay scatter plots of the *R*^2^ values among the linked SSR pairs vs. the physical distance (Mb) between those markers were generated. The LD decay was calculated as previously described [71].

### Regional association mapping

Both the Q matrix and K matrix were calculated using allelic data from 93 SSR markers (Additional file 1: Table S7) with single banding patterns [72] that were evenly distributed across all 19 rapeseed chromosomes. The population structure (Q matrix) was determined using STRUCTURE v2.2 [73]. Then five independent simulations were processed for each k, ranging from 1 to 20, with a 100,000 burn-in length and 100,000 iterations as previously reported [74]. The kinship matrix K was estimated using TASSEL 3.0 [10,75,76].

We performed regional association mapping using 17 SSR loci within the target QTL genomic regions. The association analysis was calculated using the mixed linear model (MLM) method [76] incorporated into the TASSEL 3.0 software. The significant marker-trait associations were declared for P ≤ 0.01. The conditional analysis was also calculated by TASSEL 3.0 in the MLM model using one trait as a covariate for another trait.

## Abbreviations

SW: Seed weight; SWm: Main raceme thousand seed weight; SWb: Raceme branch thousand seed weight; SWw: Whole-plant thousand seed weight; SL: silique length; QTL: Quantitative trait locus; NIL: Near isogenic lines; MAGIC: Multi-parent advanced generation inter-crosses; NAM: Nested association mapping; LD: Linkage disequilibrium; GWA: Genome-wide association; SSR: Single sequence repeat; STS: Sequence tagged site; SNP: Single nucleotide polymorphism; InDel: Insertion /Deletion; cM: Centimorgans; MLM: Mixed linear model.

## Competing interests

The authors declare no competing financial interests.

## Authors’ contributions

Conceived and designed the experiments: JQS, HZW; performed the experiments: NL, JQS; analyzed the data: NL, JQS; contributed reagents/materials/analysis tools: HZW, GHL, XFW; wrote the manuscript: NL JQS. All authors read and approved the final manuscript.

## Supplementary Material

Additional file 1: Table S1 Descriptive statistics of seed weight (g) and silique length (mm) in the linkage and association populations. **Table S2.** ANOVA and broad-sense heritability (*h*^*2*^) of seed weight and silique length. **Table S3.** Correlation coefficients of seed weight in different tissues in the same experiment. **Table S4.** The constructed genetic linkage map for the F_2_ population. **Table S5.** Identified QTLs for seed weight and silique length. **Table S6.** Molecular markers (within the target QTLs regions) used for association mapping. **Table S7.** Markers used for population structure analysis. **Table S8.** Genotype of the 17 SSR loci in association population. **Table S9.** Family relatedness for the association population. **Table S10.** Effect estimates of the three co-localized SW and SL QTLs in the linkage population. Click here for file

Additional file 2: Figure S1 Details of the climate conditions, including monthly mean temperature, monthly maximum temperature, monthly minimum temperature, monthly sunshine and monthly rainfall during the growing season. Click here for file
