# Supplementary figures and images for: A combined linkage and regional association mapping validation and fine mapping of two major pleiotropic QTLs for seed weight and silique length in rapeseed (Brassica napus L.)
Source: BMC Plant Biol. 2014 Apr 29;14:114. doi: 10.1186/1471-2229-14-114 (PMC4021082; doi:10.1186/1471-2229-14-114)

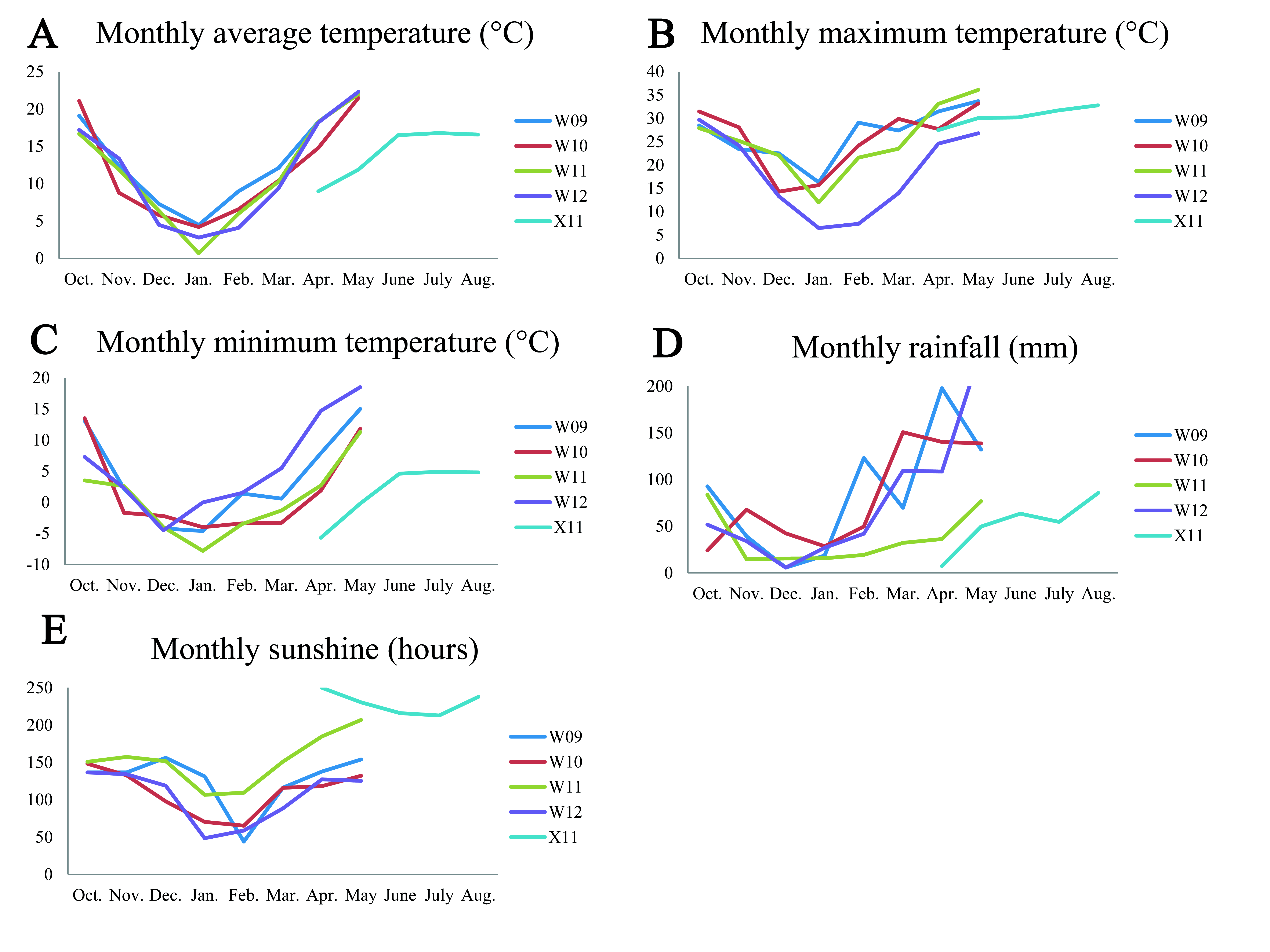

Supplement: Additional file 2: Figure S1 — Details of the climate conditions, including monthly mean temperature, monthly maximum temperature, monthly minimum temperature, monthly sunshine and monthly rainfall during the growing season. [file 1471-2229-14-114-S2.tiff]
